# Serum Ghrelin; A New Surrogate Marker of Gastric Mucosal Alterations in Upper Gastrointestinal Carcinogenesis

**DOI:** 10.1371/journal.pone.0074440

**Published:** 2013-09-30

**Authors:** Alireza Sadjadi, Abbas Yazdanbod, Yeong Yeh Lee, Majid Boreiri, Fatemeh Samadi, Behrooz Z. Alizadeh, Farhad Islami, Valerie Fyfe, Masoud Babaei, Mohammad J. Namazi, James J. Going, Masoud Sotoudeh, Geertruida H. de Bock, Reza Malekzadeh, Mohammad H. Derakhshan

**Affiliations:** 1 Digestive Disease Research Center, Tehran University of Medical Sciences, Tehran, Iran; 2 Department of Epidemiology, University of Groningen, University Medical Center Groningn, Groningen, The Netherlands; 3 Gastrointestinal Cancer Research Center, Ardabil University of Medical Sciences, Ardabil, Iran; 4 Institute of Cardiovascular & Medical Sciences, University of Glasgow, Glasgow, United Kingdom; 5 School of Medical Sciences, Universiti Sains Malaysia, Kubang Kerian, Kelantan, Malaysia; 6 Institute for Translational Epidemiology, Mount Sinai School of Medicine, New York, United States of America; 7 Sabzevar University of Medical Sciences, Sabzevar, Iran; 8 Institute of Cancer Sciences, University of Glasgow, Glasgow, United Kingdom; Monash University, Australia

## Abstract

**Background:**

A few studies have indicated inverse relationships between serum ghrelin and gastric and esophageal cancers but those associations have been restricted to specific populations, including smokers and overweight individuals. We examined the association between ghrelin and gastroesophageal cancers and atrophic gastritis in a population-based setting.

**Methods:**

In total 220 gastroesophageal cancers, comprising non-cardia and cardia gastric cancer, esophageal adenocarcinoma, esophageal squamous cell carcinoma (SCC) and age and gender-matched controls were recruited. Serum ghrelin, pepsinogen I/II ratio (PGI/II) and anti-*H.pylori* IgG antibodies were measured. Relationships between ghrelin and gastroesophageal cancers, after adjustment for PGI/II ratio, *H.pylori* status and smoking, were tested using logistic regression. Furthermore, in 125 endoscopically normal volunteers, with and without histological atrophic gastritis, the relationship with ghrelin was compared.

**Results:**

Serum ghrelin (lowest vs. highest quintile) was inversely associated with gastric cancer: OR (95% CI) 8.71 (1.70–44.59) for cardia and 6.58 (1.26–34.46) for non-cardia cancer. Lower serum ghrelin was also associated with esophageal SCC: OR (95% CI) 5.69 (1.36–23.78), but not with esophageal adenocarcinoma. A similar association was observed between gastric cancer (cardia and non-cardia) and esophageal SCC when serum ghrelin was analysed as a continuous scaled variable. In endoscopically-normal volunteers, extensive atrophic gastritis was associated with low serum ghrelin [OR (95% CI) 0.25 (0.10–0.64)].

**Conclusion:**

Inverse associations between ghrelin and some gastroesophageal cancers suggest a potential role for serum ghrelin as a biomarker of upper gastrointestinal cancers and atrophic gastritis. In areas with a high incidence of gastric and/or esophageal cancer, screening might be more effectively targeted to individuals with low serum ghrelin in addition to the PGI/II ratio.

## Introduction

Gastric and esophageal cancers are among the most prevalent malignancies worldwide, claiming more than 1,000,000 lives annually [1]. In gastric adenocarcinoma, long-term mucosal damage associated with *Helicobacter pylori* (*H.pylori*) infection results in a cascade of atrophic gastritis, intestinal metaplasia, and dysplasia leading to cancer [2]. In the process of gastric carcinogenesis, hypochlorhydria, is caused by loss of parietal cells in atrophic gastritis and severe chronic inflammation of the gastric body mucosa [3], [4]. Concurrent loss of chief cells from deeper gastric glands may lead to a reduction locally and in the serum of pepsinogen I (PGI) and pepsinogen II (PGII). The serum PGI and to a lesser extent PGII decrease during the process of atrophic gastritis, which prompts the use of serum PGI/II ratio as a surrogate marker of atrophic gastritis and cancer risk [5].

As well as hydrochloric acid and pepsinogen, the gastric mucosa secretes several other biologically active peptides and hormones. Notably, ghrelin is a 28-amino acid peptide hormone [6], produced predominantly by the P/D1 cells of gastric oxyntic gland and is mainly found in the proximal stomach [7]. Ghrelin has many biological activities, having both autocrine and paracrine roles, in the regulation of appetite and gut motility, regulation of growth hormone release and immunomodulation [8], [9].

The functional state of the gastric mucosa and the presence of *H.pylori* infection are important for carcinogenesis of both cardia and non-cardia cancers [10], [11]. Recent studies indicate altered ghrelin expression in patients with in upper gastrointestinal cancers, and its lower level in gastric atrophy [12] suggests that it is a marker of gastric mucosal function. Three recent studies have reported an inverse relationship between serum ghrelin and the risk of upper gastrointestinal cancers [13], [14], [15]. However, two of these studies nested in the Alpha-Tocopherol Beta-Carotene Cancer Trial (ATBC) were restricted to male smokers [13], [15], and in the third study, an inverse relationship between serum ghrelin and esophageal adenocarcinoma only seen in an overweight subgroup of subjects [14].

In this study, we aimed to investigate relationship between serum ghrelin and four common upper gastrointestinal cancers, namely gastric (non-cardia and cardia) adenocarcinoma, esophageal adenocarcinoma and esophageal squamous cell carcinoma (SCC), all in a population from the Ardabil Province, Iran known to have a high incidence of gastric cancer [16]. In addition to test the hypothesis that ghrelin-related carcinogenesis could be linked to atrophic gastritis, associations between various degrees of atrophic gastritis and serum ghrelin were investigated in a group of endoscopically normal volunteers.

## Materials and Methods

### Ethics statements

All subjects including potential controls gave written informed consent according to the declaration of Helsinki. The Aras Gastrointestinal Health Survey and the current case-control study were both approved by the Medical Ethics Committee of Tehran University of Medical Sciences.

### Study design and setting

Patients with upper gastrointestinal cancer (cases) and controls were drawn from a large gastroesophageal health survey, conducted in Aras Clinic in Ardabil province north-western Iran. Aras Clinic is a regional referral centre for the investigation, treatment and prevention of upper gastrointestinal tract disease throughout Ardabil province, a well-known high-risk region for gastric cancer [16], [17]. The present study used stored serum from controls and patients diagnosed between 2005 and 2007.

Prior to endoscopy, the investigators conducted a standardized interview with each patient and details of his/her lifestyle factors were systematically documented. History of smoking was recorded as numbers of cigarettes smoked per day and duration of smoking in years. A fasting venous blood sample was collected from each patient before endoscopy and the serum was stored at –70°C for later serologic assessment.

A frequency-matched case control design was used with one control for each cancer case. Controls were selected at random from a contemporaneous group of endoscopy negative dyspeptic patients with no identifiable peptic ulceration, gastroduodenal erosions or tumors. Controls matched for gender, residence place and age ±4 years were interviewed for detailed lifestyle similarly to cases and had their serum stored prior to endoscopy.

### Upper gastrointestinal cancers

Of 247 patients with gastric or esophageal cancer identified during the health survey, 27 (11%) were excluded due to insufficient or inappropriate serum or histological samples. Therefore, the laboratory and questionnaire data for the remaining 220 (72 non-cardia gastric cancers, 53 cardia gastric cancer, 36 esophageal adenocarcinoma and 59 esophageal SCC) were analyzed in the current study. All diagnoses of gastric or esophageal cancer were confirmed histologically through endoscopic and/or surgical biopsy. All histological sections were examined by experienced gastrointestinal pathologists and reviewed by a lead pathologist (M.S). All pathological diagnoses met protocol requirements in accordance with International Classification of Disease, Oncology, version 3 (ICD-O-3) [18]. In difficult cases, a diagnosis of cancer was made only after joint review by the panel of pathologists. The following ICD-O-3 codes were used to categorize the four cancer groups: for esophageal squamous cell carcinoma, topography C15.0– C15.9, and morphology M8070/3; for esophageal adenocarcinoma, topography C15.0– C15.7, and morphology M8140/2, M8140/3; for gastric cardia and esophagogastric junctional cancer topography C16.0 and morphology M8140/2, M8140/3, M8144/3, and M8145/3; for gastric non-cardia cancer topography C16.1– C16.7, and morphology M8140/2, M8140/3, M8144/3, and M8145/3. The histological subtype of gastric adenocarcinoma was classified as intestinal, diffuse or mixed subtypes according to the original classification introduced by Lauren [19].The similar classification was applied to esophageal adenocarcinoma.

### Atrophic gastritis

To investigate further relationship between serum ghrelin and precancerous changes in the stomach, a group of endoscopically normal volunteers (n = 125) were recruited in a consecutive fashion and divided into three histologically-defined groups. These histological groups, age and gender-balanced, were as following: without atrophic gastritis (n = 46), with mild-moderate antral atrophy (n = 29) and with extensive body and antral atrophy (n = 50). Atrophic gastritis was defined as loss of glandular tissue in the gastric mucosa and scored based on a semi-quantitative scale recommended by the Updated Sydney System for classification and grading of gastritis [20]. Serum ghrelin and pepsinogen measurement were performed in these three groups with method similar to that applied to main study subjects.

### Laboratory Assessment

Aliquots of serum stored at −70 centigrade stored from cases and controls were analyzed for total serum ghrelin, PGI, PGII and anti *H.pylori* IgG antibodies. Total serum ghrelin was quantified by radioimmunoassay using a polyclonal rabbit antibody from Mediagnost (Reutlingen, Germany), which assesses the peptide from its c-terminus, thus allowing valid measurements over longer periods of preservation [21]. Ghrelin measurements were performed according to the kit manufacturer's instructions, on serum specimens without any treatment. Sensitivity of the assay was 40 pg/ml measured as 2× standard deviation of zero standards. Manufacturer's data indicate maximum intra-assay and inter-assay coefficients of variation of 5.3% and 8.2%, respectively. Serum PGI and PGII were assayed by an enzyme immuno-sorbant assay using monoclonal antibodies to PGI and PGII (BIOHIT diagnostics, Biohit Ltd, UK) according to the manufacturer's instructions. PGI and PGII values were reported in µg/L. The PGI/II ratio was calculated and reported as a dimensionless fraction. *H.pylori* status of cases and controls was assessed by ELISA using anti-*H.pylori* IgG antibody, also from BIOHIT diagnostics, Biohit Ltd, UK. A response titre >30 enzyme-immunounits (EIU) was considered serological evidence of *H.pylori* infection. A patient was considered infected with *H.pylori* if at least one of the three diagnostic tests (rapid urease test, histology and serology) was positive.

### Data and Statistical Analysis

The measured values of total serum ghrelin were reported in two formats: as quintiles (1^st^ to 5^th^ quintiles) and as a continuous variable. To generate scaled continuous variable of ghrelin, the interquartile range of ghrelin in each control group was determined, and half of the difference was calculated [scaling factor  =  (quartile 75%– quartile 25%)/2]. The ghrelin values for all cases in each of the four cancer groups and their controls were divided by the relevant scaling factor. Using binary logistic regression, the relationship between each ghrelin quintile and the four cancer groups was estimated and reported as odds ratio (OR), 95% confidence interval and related *p* values. In all models containing serum ghrelin quintiles, the 5^th^ quintile (last) was set as the reference group. In the multivariable model, serum PGI/II ratio, history of smoking, and *H.pylori* status were used as potential confounders. *H.pylori* infection and status of smoking (0 =  Never, 1 =  smoking of ≥1 cigarettes per day for at least 10 years but no more than 5 years have passed since stopping, in the case of an ex-smoker) were analyzed as a dichotomous variable. The difference in ghrelin and PGI/II ratio levels between groups having different grades of atrophic gastritis was tested using the Mann-Whitney U test. Two sided *P* values <0.05 were considered statistically significant. Statistical analyses were conducted using SPSS statistical package version 19 (IBM Corp., NY, USA).

## Results

The main characteristics of the four groups of patients with upper gastrointestinal cancers and their matched control groups are summarised in Table 1.

**Table 1:** Frequency of studied risk factors in cases and their matched controls for gastric and esophageal cancers by anatomical and histological classification.

	PG I/II ratio	Smoking	*H.pylori*	Histological Subtype		
	<2.5	(Ever)	Infection	Intestinal	Diffuse	Mixed
**Gastric non-cardia**						
Cases (n = 72)	69.9%	33 (45.8%)	68 (94.4%)	42 (58.3%)	25 (34.7%)	5 (6.9%)
Controls (n = 72)	29.6%	20 (27.8%)	62 (86.1%)	n/a	n/a	n/a
**Gastric cardia**						
Cases (n = 53)	47.2%	19 (35.8%)	44 (83.0%)	34 (64.1%)	16 (30.2%)	3 (5.7%)
Controls (n = 53)	22.6%	14 (26.4%)	46 (86.8%)	n/a	n/a	n/a
**Esophageal Adenocarcinoma**						
Cases (n = 36)	11.1%	19 (52.8%)	23 (63.9%)	31 (86.1%)	2 (6.6%)	3 (8.3%)
Controls (n = 36)	27.8%	9 (25.0%)	31 (86.1%)	n/a	n/a	n/a
**Esophageal squamous cell carcinoma**						
Cases (n = 59)	49.2%	27 (45.7%)	56 (94.9%)	n/a	n/a	n/a
Controls (n = 59)	23.7%	16 (27.1%)	53 (89.9%)	n/a	n/a	n/a

### Gastric non-cardia cancer

A total of 72 patients (54 males and 18 females, mean age 67.9±6.4 years) with non-cardia cancer and 72 controls were studied. Histologically, 58.3% (42/72) of cancers were of intestinal subtype, 34.7% (25/72) of diffuse subtype and 6.9% (5/72) of mixed subtype. An ascending pattern in the risk of cancer was observed from the highest to the lowest quintiles of serum ghrelin levels (Table 2). Cancer risk was maximum in patients having ghrelin levels in the 1^st^ quintile (≤570 pg/ml) with OR  = 12.01 (95% CI: 2.99–48.25) compared to patients in the 5^th^ quintile. The inverse association observed between serum ghrelin (1^st^ vs. 5^th^ quintile) and non-cardia gastric cancer diminished in strength but remained statistically significant after adjustment for serum PGI/II ratio, smoking and *H.pylori* infection status, with OR of 6.58 (95% CI: 1.26–34.46). The continuous variable of ghrelin (scaled to 159 pg/ml) supported the inverse relationship observed between serum ghrelin and non-cardia gastric cancer, with OR (95% CI)  = 2.09 (1.53–2.84) and 1.81 (1.25–2.64) for decreasing level of ghrelin before and after adjustment for serum PG I/II ratio, smoking and *H.pylori* infection status, respectively.

**Table 2:** Risk of gastric non-cardia cancer associated with serum ghrelin presented as either quintiles or continuous variable before and after adjustment for serum PG I/II ratio, *H.pylori* status and smoking.

			Non-adjusted			Adjusted		
	Cases	Controls	OR	95% CI	*P* value	OR	95% CI	*P* value
	n (%)	n (%)						
**Ghrelin Quintiles** (pg/ml)								
5^th^: 960–1098	3 (4.2%)	14 (19.4%)	1.00			1.00		
4^th^: 824–957	5 (6.9%)	15 (20.8%)	1.56	0.31–7.75	0.691	1.06	0.17–6.81	0.952
3^rd^: 708–818	10 (13.9%)	14 (19.4%)	3.33	0.75–14.76	0.113	2.65	0.49–14.35	0.257
2^nd^: 579–693	17 (25.0%)	15 (20.8%)	5.60	1.35–23.23	0.018	3.63	0.70–18.78	0.124
1^st^: 246–570	36 (50.0%)	14 (19.4%)	12.01	2.99–48.25	<0.001	6.58	1.26–34.46	0.026
**Decreasing ghrelin as continuous variable** (scaled to 159 pg/ml)			2.09	1.53–2.84	<0.001	1.81	1.25–2.64	0.002

### Gastric cardia cancer

Fifty-three patients (37 males and 16 females, mean age 63.9±7.1 years) with cardia cancer and similar number of controls were included in the study. Of histological subtypes, 64.2% (34/53) of cancers were of intestinal subtype, 30.2% (16/53) of diffuse subtype and 5.7% (5/53) of mixed or undifferentiated subtype. Similar to non-cardia cancer but to a lesser extent, an increasing cancer risk was observed with decreasing levels of ghrelin (Table 3). The risk was maximum in patients having ghrelin levels in the 1^st^ quintile (≤647 pg/ml) than patients in the 5th quintile (984–1158 pg/ml), with OR = 6.00 (95% CI: 1.52–23.71). Multivariable analysis, after adjustment for serum PGI/II ratio, smoking and *H.pylori* infection, strengthened the inverse association observed between serum ghrelin (1^st^ vs. 5^th^ quintile) and cardia cancer, with OR (95% CI)  = 8.71 (1.70–44.59). Likewise, the continuous variable of ghrelin, scaled to 122 pg/ml, demonstrated a positive relationship between decreasing level of serum ghrelin and gastric cardia cancer, with OR (95 CI%)  = 1.64 (1.25–2.15) and 1.75 (1.24–2.47), before and after adjustment for serum PGI/II ratio, smoking and *H.pylori* infection status, respectively.

**Table 3:** Risk of gastric cardia cancer associated with serum ghrelin presented as either quintiles or continuous variable before and after adjustment for serum PG I/II ratio, *H.pylori* status and smoking.

			Non-adjusted			Adjusted		
	Cases	Controls	OR	95% CI	*P* value	OR	95% CI	*P* value
	n (%)	n (%)						
**Ghrelin Quintiles** (pg/ml)								
5^th^: 984–1158	4 (7.5%)	10 (18.9%)	1.00			1.00		
4^th^: 869–976	4 (7.5%)	11 (20.8%)	0.91	0.18–4.64	0.909	0.99	0.18–5.47	0.990
3^rd^: 778–864	7 (13.2%)	11 (20.8%)	1.59	0.36–7.11	0.543	2.20	0.45–10.87	0.334
2^nd^: 648–776	14 (26.4%)	11 (20.8%)	3.18	0.78–12.94	0.106	5.91	1.19–29.28	0.030
1^st^: 249–647	24 (45.3%)	10 (18.9%)	6.00	1.52–23.71	0.011	8.71	1.70–44.59	0.009
**Decreasing ghrelin as continuous variable** (scaled to 122 pg/ml)			1.64	1.25–2.15	0.001	1.75	1.24–2.47	0.002

### Esophageal Adenocarcinoma

There were 36 patients with esophageal adenocarcinoma with mean age of 63.4 (±4.2) years and an obvious pattern of male-predominance (26 males and 10 females). Histologically, 92% (33/36) were of intestinal subtype, except for two cases of diffuse subtype and one mixed subtype. None of the serum ghrelin quintiles were associated with esophageal adenocarcinoma in either univariable or multivariable analysis, but there was marginal inverse relationship seen between decreasing ghrelin level as a continuous variable and risk of esophageal adenocarcinoma, being OR (95% CI) of 0.65 (0.43–1.01) and 0.60 (0.35–1.04) in univariable and multivariable analyses, respectively (Table 4).

**Table 4:** Risk of esophageal adenocarcinoma associated with serum ghrelin presented as either quintiles or continuous variable before and after adjustment for serum PG I/II ratio, *H.pylori* status and smoking.

			Non-adjusted			Adjusted		
	Cases	Controls	OR	95% CI	*P* value	OR	95% CI	*P* value
	n (%)	n (%)						
**Ghrelin Quintiles** (pg/ml)								
5^th^: 995–1208	10 (27.8%)	7 (19.4%)	1.00			1.00		
4^th^: 904–992	9 (25.0%)	7 (19.4%)	0.90	0.23–3.58	0.881	0.31	0.05–1.76	0.185
3^rd^: 789–889	9 (25.0%)	8 (22.2%)	0.79	0.20–3.06	0.730	0.72	0.14–3.81	0.697
2^nd^: 631–783	6 (16.7%)	7 (19.4%)	0.60	0.14–2.58	0.492	0.41	0.06–2.67	0.352
1^st^: 306–617	2 (5.6%)	7 (19.4%)	0.20	0.03–1.27	0.087	0.11	0.01–1.15	0.066
**Decreasing ghrelin as continuous variable** (scaled to 161 pg/ml)			0.65	0.43–1.01	0.053	0.60	0.35–1.04	0.068

### Esophageal Squamous Cell Carcinoma

There were 59 patients (36 males and 23 females) with esophageal squamous cell carcinoma and their mean (SD) age was 65.7 (5.3) years. Anatomically, 58% (34/59) of tumors were located in the middle third, 32% (19/59) in the upper third and only 10% (6/59) in the lower third of esophagus. Serum ghrelin level was inversely associated with presence of cancer, with the highest occurrence being observed in patients with ghrelin levels in the 1^st^ quintile versus those in the 5^th^ quintile, OR (95% CI)  = 5.96 (1.57–22.60). The cancer risk remained essentially unchanged after adjustment for serum PGI/II ratio, *H.pylori* infection and smoking. With ghrelin as continuous variable, a similar positive association was observed between decreasing level of ghrelin and esophageal squamous cell carcinoma with both univariable and multivariable analyses (Table 5).

**Table 5:** Risk of esophageal squamous cell carcinoma associated with serum ghrelin presented as either quintiles or continuous variable before and after adjustment for serum PG I/II ratio, *H.pylori* status and smoking.

			Non-adjusted			Adjusted		
	Cases	Controls	OR	95% CI	*P* value	OR	95% CI	*P* value
	n (%)	n (%)						
**Ghrelin Quintiles** (pg/ml)								
5^th^: 966–1098	4 (6.8%)	12 (20.3%)	1.00			1.00		
4^th^: 847–963	5 (8.5%)	12 (20.3%)	1.15	0.24–5.39	0.863	1.52	0.28–8.18	0.629
3^rd^: 770–846	7 (11.9%)	12 (20.3%)	1.60	0.37–7.02	0.530	1.71	0.35–8.34	0.510
2^nd^: 644–769	17 (28.8%)	12 (20.3%)	3.90	1.00–15.21	0.050	4.69	1.03–21.46	0.046
1^st^: 246–638	26 (44.1%)	11 (18.6%)	5.96	1.57–22.60	0.009	5.69	1.36–23.78	0.017
**Decreasing ghrelin as continuous variable** (scaled to 141 pg/ml)			1.80	1.32–2.46	<0.001	1.78	1.275–2.48	0.001

### Ghrelin and Atrophic Gastritis

**Figure 1:**
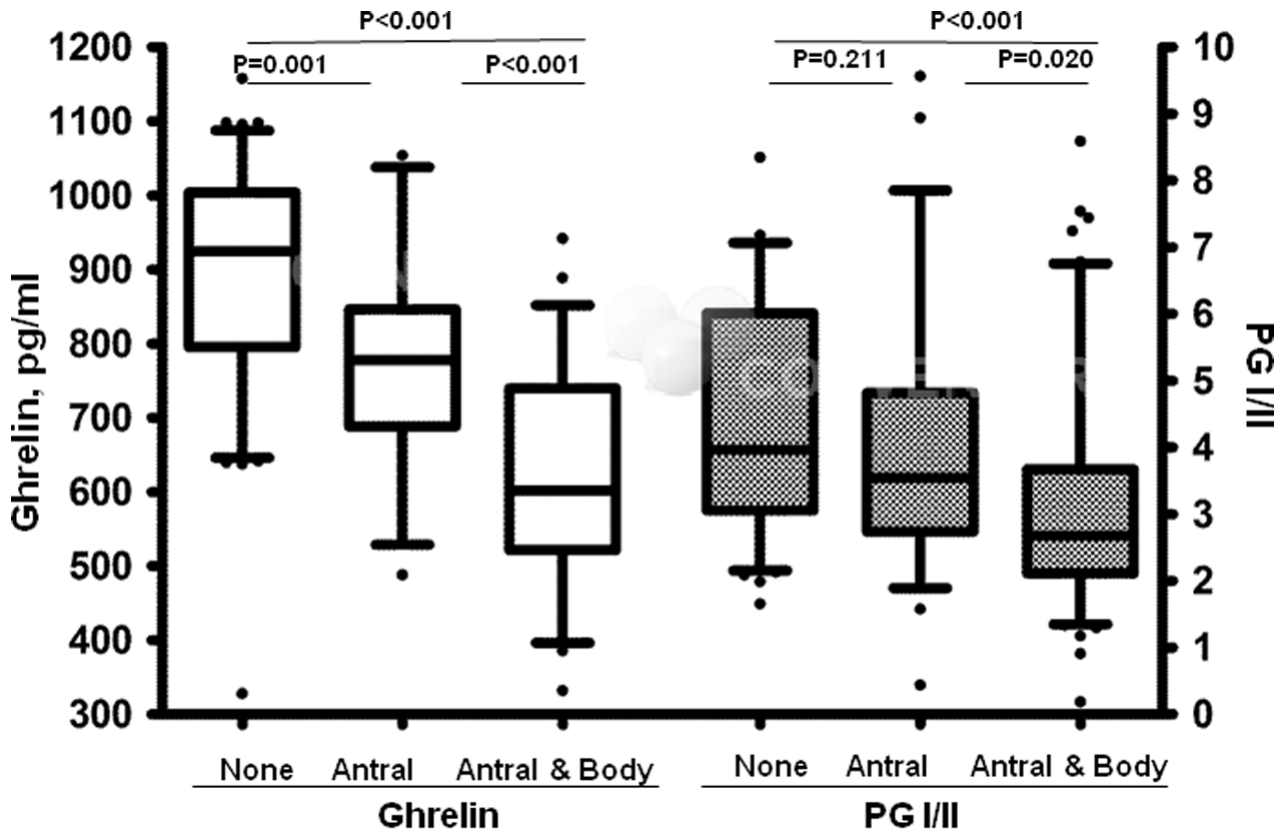
Association between histological atrophic gastritis limited to antrum (antral) or extended to body (extensive) and serum ghrelin versus serum PGI/II ratio, all statistical comparisons tested by Mann-Whitney U test.

Total serum ghrelin was highest in the group without atrophic gastritis (Med:925, IQR:209), lowest in the group with extensive atrophic gastritis (Med:602, IQR:218) and antral atrophic gastritis (Med:778, IQR:158) in-between the two. Both groups with atrophy (antral and extensive) had significantly lower ghrelin level compared to the non-atrophic group (p values <0.01). Similar to ghrelin, PGI/II ratio was also highest in the group without atrophy, and lowest in the group with extensive atrophy but the difference between non-atrophic and antral atrophic groups was not statistically significant (p value = 0.211) (Figure 1). Furthermore, total serum ghrelin was correlated with serum PGI/II ratio, but the magnitude of the correlation was not large (CC = 0.40, p value <0.01).

Logistic regression model was used to compare the magnitude of association between extensive atrophic gastritis and either scaled ghrelin or PGI/II ratio. Between the two variables, scaled ghrelin but not PGI/II ratio showed a significant inverse association with extensive atrophic gastritis [(OR = 0.254, 95%CI: 0.101–0.638), p value = 0.004].

## Discussion

In this case-control study, we found statistically significant inverse relationships between serum ghrelin and gastric non-cardia cancer, gastric cardia cancer and esophageal squamous cell carcinoma (SCC). The relationship between ghrelin and gastric adenocarcinoma, however, was not statistically significant. Moreover, we found that cancer-free individuals with extensive atrophic gastritis had significantly lower serum ghrelin.

Only a few published studies examined relationships between serum ghrelin levels and gastric [15], [22] or esophageal [13], [14] cancer. All of these studies required further investigations in different populations to confirm their findings, and had limitations in sample size. The current study provides robust evidence of the role of serum ghrelin level, as a surrogate marker of gastric mucosal function, in three common types of upper gastrointestinal cancers in a high-risk population residing in Ardabil province, Iran.

In the current study, both in quintiles and scaled continuous variable, serum ghrelin levels were observed to have an inverse relationship with the risk of non-cardia gastric cancer. This is consistent with findings of Murphy *et al.* [15], the only study in which the location of non-cardia gastric cancer has been clearly defined. The negative association between serum ghrelin level and non-cardia gastric cancer remained significant after adjustments for PGI/II ratio, smoking and *H.pylori* infection, indicating the independent nature of the observed inverse association.

The mechanism underlying the inverse relationship between non-cardia gastric cancer and serum ghrelin levels has not been thoroughly investigated by previous studies. Ghrelin is produced by several different tissues in the human body (including hypothalamus, pituitary, small and large intestine and pancreas), but its main source is the gastric oxyntic epithelium, largely in unacetylated form [7]. The ghrelin immunoreactive cells are located close to parietal cells [23], and atrophic gastritis is thought to involve loss of ghrelin-producing cells. This hypothesis has been supported by studies showing lower levels of ghrelin in patients with atrophic gastritis [24]–[25]. A strong association between ghrelin and extensive gastric atrophy in our study supports the suggested mechanism. The profound and long-term reduction of the serum ghrelin following resective gastric bypass is another evidence of gastric body mucosa serving as the main source of ghrelin which is eliminated by surgical resection rather than atrophic gastritis [26]. Moreover, long standing chronic inflammation of gastric mucosa with or without atrophic changes during *H.pylori* infection is associated with considerable reduction of fundic ghrelin mRNA expression and plasma ghrelin concentration [27], [28]. In patients with more advanced gastric cancer, several factors including ghrelin, leptin, adiponectin and insulin-like growth factor -I (IGF-I) are involved in the cachexia [29].

As with non-cardia cancers, an increasing risk of gastric cardia cancer with decreasing levels of serum ghrelin was also observed. Patients with gastric cardia cancers in our study are in part comparable to patients with esophago-gastric junction cancer in the study by Murphy *et al*. [15], but tumors of the distal esophagus are not covered by our definition of cardia cancer. The relatively weaker inverse relationship of ghrelin level in cardia vs. non-cardia gastric cancer is consistent with the null association observed between esophageal adenocarcinoma and ghrelin levels in our study.

The absence of a statistically significant relationship between serum ghrelin and esophageal adenocarcinoma was another important finding. In a nested case-control study by de Martel *et al*. [14], a very high concentration (>3200 pg/ml) of serum ghrelin was associated with a lower risk of esophageal adenocarcinoma, a finding was unexpected even to the authors themselves. They realized that the inverse association is evident only in overweight subjects with body mass index (BMI) >25. We attempted to replicate their subgroup analysis, but due to the retrospective nature of our study, BMI data was only available in half of the cancer cases, all with BMI between 20.5 and 24.6 except for one with BMI of 25.8 (analysis not shown). Therefore, our observations and that of de Martel's study should be interpreted with caution since it may not represent the real nature of association between ghrelin levels and esophageal adenocarcinoma. In addition, considering marked inverse association between esophageal adenocarcinoma and atrophic gastritis [30], the lack of association (or a non-significant inverse association) between esophageal adenocarcinoma and serum ghrelin in the current study is an expected finding.

In order to improve our understanding of ghrelin's association with a wide range of upper gastrointestinal cancers, we included a group of patients with esophageal squamous cell carcinoma. The inverse relationship between ghrelin levels, both in quintiles and in continuous variable, with squamous cell carcinoma was evident in both univariable and multivariable analyses. Similar results were reported by Murphy *et al*. [13]; however in their study, they have only adjusted the association to PGI but not PGI/II ratio, as a marker of atrophic gastritis.

Many studies [31]–[38] have reported a greater risk of esophageal squamous cell carcinoma in subjects with atrophic gastritis. Although serum PGI/II ratio has been used as a surrogate marker of atrophic gastritis in most studies [31]–[36], the positive association between the two has been confirmed even if the presence of atrophic gastritis is determined by histological [37] or endoscopic methods [38], indicating robustness of the association.

If the inverse relationship between serum ghrelin and esophageal squamous cell carcinoma is attributed to the presence of atrophic gastritis, why is the association independent from PG/II ratio in our study and from PGI in Murphy's [13] study? Both serum ghrelin and PGI/II ratio (and PGI to a lesser extent) are serological markers of atrophic gastritis, but they may not represent the same changes during the process of oxyntic glands destruction in atrophic gastritis, because they are produced by different cell types at different depths within the oxyntic glands. An unknown degree of sampling error and inaccurate measurement due to potential circadian rhythm, in the case of serum PGI/II ratio and even serum ghrelin may explain their independent association with the atrophic gastritis in the process of cancer genesis. Moreover, exact mechanisms responsible for the excess risk of esophageal squamous cell carcinoma in subjects with atrophic gastritis are not clear, although high levels of nitrosamine compounds or other carcinogenic products of bacterial overgrowth in the stomach have been proposed as a potential pathway [39].

The results of the current study provide evidence for serum ghrelin being a surrogate marker of gastric mucosal function in upper gastrointestinal cancers in a population setting different from the other three studies mentioned above [13], [14], [15]. The homogenous distribution of *H.pylori* infection and smoking rates in the control group and their similarity to general residents of the target area allowed them being acceptable representatives of the general population [40]–[41]. Our study does have some limitations, primarily the small number of esophageal adenocarcinoma cases, deficient information on BMI and addressing limited number of environmental risk variables. Additional measurement of acylated vs. unacylated forms of ghrelin may be ideal due to their differential effect and this should be considered in the future study design.

In conclusion, further evidence presented in the current comprehensive study supports the role of ghrelin as a marker of gastric mucosal alterations in upper gastrointestinal cancers in a population who are known to be at high risk of gastric cancer. These results provide further insight into the pathogenesis of upper gastrointestinal cancers, and may provoke more detailed investigations leading to identification of a panel of diagnostic serological markers applicable to surveillance programmes.
